# In silico ADME predictions and in vitro antibacterial evaluation of 2-hydroxy benzothiazole-based 1,3,4-oxadiazole derivatives

**DOI:** 10.3906/kim-1912-55

**Published:** 2020-08-18

**Authors:** Afnan Ahmed ALGHAMDI, Mohammad Mahboob ALAM, Syed NAZREEN

**Affiliations:** 1 Department of Chemistry, Faculty of Science, Albaha University, Albaha Saudi Arabia

**Keywords:** Heterocycles, pharmacokinetics, minimum inhibitory concentration

## Abstract

In the present work, a library of fifteen 2-hydroxy benzothiazole-linked 1,3,4 -oxadiazole derivatives have been synthesized and confirmed using different analytical techniques. All of the synthesized compounds have been tested for antibacterial and in silico pharmacokinetic studies for the first time. From the ADME predictions, compound 4 showed the highest in silico absorption percentage (86.77%), while most of the compounds showed more than 70% absorption. All of the compounds comply with the Lipinski rule of 5, suggesting that the compounds possess good drug likeness properties upon administration. Furthermore, all of the compounds follow the Veber rule, indicating good bioavailability and good intestinal absorption. The antibacterial results exhibited excellent to moderate activity. Compounds **5** , **9** , **12** , **14** , **15** , **16** , and **17** were the most active compounds against the tested bacterial strains. Compound **14** showed comparable MIC 6.25 ±0.2 μg/disc to the standard drug amoxicillin against the tested Gram-positive bacterial strains. Compounds **5** , **14** , **17** exhibited MIC 12.5 ±0.8 μg/disc, which was comparable to the standard drug against *E. faecalis* . It can be concluded that the synthesized compound could be used as a lead molecule in the development of new antibacterial agents with high efficacy.

## 1. Introduction

Despite advances in the development of antimicrobial drugs, microbial infections remain a great threat to humankind [1]. Increased acquired resistance in pathogenic microbial strains toward antimicrobial drugs causes ineffective treatment and persistence of infections, which sometimes leads to death [2]. Therefore, there is need to develop cost-effective and potent new antimicrobial agents for the treatment of resistant pathogenic microbial strains.

In recent years, heterocyclic compounds have emerged as a potent scaffold in medicinal chemistry, as they influence the properties of a drug in terms of lipophilicity, solubility, and so on [3]. Benzothiazole is an important heterocyclic pharmacophore with various pharmacological properties, such as antimicrobial [4–6], anticancer [7], antidiabetic [8], antiviral [9], antimalarial [10], antihelimintic [11], anticonvulsant [12,13], and antileishmanial [14] properties. This moiety is present in many marketed drugs like ethoxzolamide, frentizole, riluzole etc. On the other hand, 1,3,4-oxadiazole moiety is also present in marketed drugs such as zibotentan, raltegravir, and nesapidil. These different drugs are used for different diseases such as diabetes, microbial infections, viral infections, tuberculosis, inflammatory problems, and cancer [15–19].

**Figure 1:**
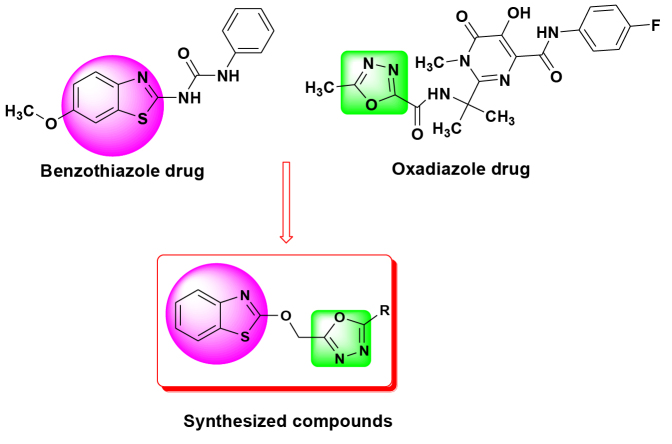
Drug based designing of the synthesized compounds.

The importance of benzothiazole and 1,3,4-oxadiazole moieties prompted us to synthesize 2-hydroxy benzothiazole-linked 1,3,4-oxadiazole derivatives (Figure 1). These derivatives were studied for in silico pharmacokinetics and drug likeness properties, which we found to be excellent. For the first time, we herein report the synthesis, pharmacokinetics, drug likeness prediction, and antibacterial activity of 2-hydroxy benzothiazolelinked 1,3,4-oxadiazole derivatives.

## 2. Materials and methods

### 2.1. Chemistry

All chemicals used in the present work were of reagent grade and were procured from Sigma-Aldrich (Hamburg, Germany) and Loba Chemie (Mumbai, India). The starting material 2-hydroxy benzothiazole ( **1** ) was purchased from Sigma-Aldrich. IR spectra were performed on a Thermo Scientific iS-50 (Thermo Fisher Scientific Inc., Waltham, MA, USA) using the ATR method. NMR spectra were performed on Bruker 300 MHz and 850 MHz instruments (Bruker Corp., Billerica, MA, USA) using the solvents CDCl_3_ or DMSO-d_6_. Tetramethylsilane (TMS) was used as an internal standard. Chemical shifts and coupling constants are provided in hertz (Hz) and parts per million (ppm), respectively. A Thermo Scientific-LCQ Fleet (LCF10605), using the electron spray ionization method, was used for recording the mass spectra, which are provided in m/z. Melting points were recorded using an automatic melting point (Stuart SMP40; Cole-Parmer, Stone, UK). Elemental analysis was performed on a LECO elemental analyzer apparatus (LECO Corp., St. Joseph, MI, USA). The elemental analysis data are reported in % standard and were within ±0.4% of the calculated values.

### 2.2. General procedure for the synthesis of compounds

#### 2.2.1. Synthesis of compound 2

In a 250-mL round bottom flask, 2-hydroxy benzothiazole (1) (10 g, 66.2 mmol), dry acetone (150 mL), and anhydrous potassium carbonate (9.0 g, 66.2 mmol) was charged. After 30 min, ethyl chloroacetate (8.07 g, 66.2 mmol) was added and refluxed for 10–12 h. The progress of the reaction was monitored by TLC. After completion of the reaction, the reaction mass was filtered; the filtrate was concentrated to 50 mL, cooled, poured on crushed ice, and extracted with dichloromethane. The dichloromethane layer was dried over anhydrous sodium sulphate, concentrated, and crystallized to produce white crystals.

#### 2.2.2. Ethyl 2-(benzo[d]thiazol-2-yloxy)acetate (2):

Yield 94%, mp. 49.8–50 °C, M.W. 237, R_f_ = 0.89 in petroleum ether/ethylacetate (6 : 4) as a developing solvent. IR (ATR, cm^-1^): 3060 (C-H aromatic), 2973 (C-H aliphatic), 1671 (C=N of benzothiazole), 1663 (C=O), 1183 (C-O of benzothiazole), 1049 (C-O), 705 (C-S). ^1^H NMR (850 MHz, DMSO-d_6_), δ (ppm): 1.21 (t, *J* =6.8 Hz, 3H, -CH_3_), 4.17 (q, *J* =6.8 Hz, 2H, -O-CH_2_ of ethyl ester), 4.84 (s, 2H, -O-CH_2-_), 7.23 (tt, *J* =0.85 Hz, 8.5 Hz, 1H, Ar-H), 7.31 (d, *J* =8.5 Hz, 1H, Ar-H), 7.36 (tt, *J* =0.85 Hz, 8.5 Hz 1H, Ar-H), 7.68 (dd, *J* = 0.85 Hz, 7.6 Hz, 1H, Ar-H). ^13^C NMR (213 MHz, DMSO-d_6_), δ (ppm): 14.03 (-CH_3_), 43.43 (-O- C H_2_2-CH_3_), 61.46 (-O-CH_2-_), Ar-C (111.41, 121.12, 123.04, 123.53 , 126.75, 136.76), 167.43 (benzothiazole, C=N), 169.21 (C=O). ESI +ve MS (m/z): 238 [M + H]^+^. Anal. Calc. for C_11_H_11_O_3_NS: C, 55.68; H, 4.67; O, 20.23; N, 5.90; S, 13.51. Found: C, 55.69; H, 4.65; O, 20.22; N, 5.91; S, 13.50.

#### 2.2.3. Synthesis of 2-(benzo[d]thiazol-2-yloxy)acetohydrazide (3)

To the solution of ethyl 2-(benzo[d]thiazol-2-yloxy)acetate ( **2** ) (8.0 g, 3.3 mmol) in ethanol (100 mL), hydrazine monohydrate (1.68 g, 3.3 mmol) was added, and the solution was refluxed for 7–8 h. After completion of the reaction monitored by TLC, the reaction mass was cooled, and the solid so obtained was filtered to give 2-(benzo[d]thioazol-2-yloxy)acetohydrazide ( **3** ) as a white solid.

#### 2.2.4. 2-(Benzo[d]thiazol-2-yloxy)acetohydrazide (3)

Yield 88%, mp. 207–208 °C, M.W. 223, R_f_ = 0.28 in petroleum ether/ethylacetate (6 : 4) as a developing solvent. IR (ATR, cm^-1^): 3311 (NH_2_), 3222 (NH), 3040 (C-H aromatic), 2986 (C-H aliphatic), 1680 (C=N of benzothiazole), 1653 (C=O), 1188 (C-O of benzothiazole), 1053 (C-O), 714 (C-S). ^1^H NMR (850 MHz, DMSO-d_6_), δ (ppm): 4.31 (s, 2H, -NH_2_), 4.56 (s, 2H, -O-CH_2-_), 7.16–7.21 (m, 2H, Ar-H), 7.34–7.35 (m, 1H, Ar-H), 7.65–7.66 (m, 1H, Ar-H), 9.45 (s, 1H, -O=C-N-H). ^13^C NMR (213 MHz, DMSO-d_6_), δ (ppm): 43.31 (-O-CH_2-_), Ar-C (111.41, 121.21, 122.83, 123.25, 126.54, 137.30), 165.40 (benzothiazole, C=N), 169.14 (C=O). ESI +ve MS (m/z): 224.00 [M + H]^+^. Anal. Calc. for C_9_H_9_O_2_N_3_S: C, 48.42; H, 4.06; O, 14.33; N, 18.82; S, 14.36. Found: C, 48.41; H, 4.05; O, 14.34; N, 18.81; S, 14.37.

#### 2.2.5. General procedure for the synthesis of 1,3,4-oxadiazole derivatives (4–18)

A mixture of 2-(benzo[d]thiazol-2-yloxy)acetohydrazide ( **3** ) (0.2 mmol) and a different substituted aromatic acid (0.2 mol) in POCl3 (10 mL) was refluxed for 8–12 h. After completion of the reactions, which were monitored by TLC, the mixture was cooled, poured onto crushed ice, and neutralized with NaHCO_3_ solution. The solid material precipitated out was filtered, washed with water, dried, and finally purified either by recrystallization with suitable solvents or column chromatography using *n* -hexane and ethylacetate as eluents.

#### 2.2.6. 2-((5-Phenyl-1,3,4-oxadiazol-2-yl)methoxy)benzo[d]thiazole (4)

Yield 57%, white, mp. 258–259 °C, M.W. 309, R_f_ = 0.64 in petroleum ether/ethylacetate (6 : 4) as a developing solvent. IR (ATR, cm^-1^): 3068 (C-H aromatic), 2956 (C-H aliphatic), 1668 (C=N of benzothiazole), 1591 (C=N of oxadiazole), 1512 (C=N of oxadiazole), 1488, 1475, 1336, 1272, 1245, 1188, 1024, 746 (C-S). ^1^H NMR (850 MHz, DMSO-d_6_), δ (ppm): 4.82 (s, 2H, -O-CH_2-_), 7.27–7.31 (m, 2H, Ar-H), 7.44–7.45 (m, 1H, Ar-H), 7.55 (t, J = 8.5 Hz, 2H, Ar-H), 7.62–7.64 (m, 1H, Ar-H), 7.73–7.76 (m, 1H, Ar-H), 7.91–7.92 (m, 2H, Ar-H). ^13^C NMR (213 MHz, DMSO-d_6_), δ (ppm): 43.25 (-O-CH_2-_), Ar-C (111.54, 121.17, 122.87, 123.38, 126.60, 127.47, 127.66, 128.54, 128.70, 131.98, 132.22, 137.11), 165.52 (oxadiazole, C=N), 165.58 (oxadiazole, C=N), 169.23 (benzothiazole, C=N). ESI +ve MS (m/z): 310 [M + H]^+^. Anal. Calc. for C_16_H_11_O_2_N_3_S: C, 62.12; H, 3.58; O, 10.34; N, 13.58; S, 10.37. Found: C, 62.10; H, 3.59; O, 10.35; N, 13.56; S, 10.36.

#### 2.2.7. 2-((5-(2-Chlorophenyl)-1,3,4-oxadiazol-2-yl)methoxy)benzo[d]thiazole (5)

Yield 54%, white, mp. 167–168 °C, M.W. 343, R_f_ = 0.62 in petroleum ether/ethylacetate (6 : 4) as a developing solvent. IR (ATR, cm^-1^): 3050 (C-H aromatic), 2986 (C-H aliphatic), 1697 (C=N of benzothiazole), 1592 (C=N of oxadiazole), 1582 (C=N of oxadiazole), 1473, 1457, 1423, 1321, 1176, 1044, 715 (C-S). ^1^H NMR (850 MHz, DMSO-d_6_), δ (ppm): 5.62 (s, 2H, -O-CH_2-_), 7.26 (t, *J* = 7.6 Hz, 1H, Ar-H), 7.41 (t, *J* = 8.5 Hz, 1H, Ar-H), 7.48 (d, *J* = 8.5 Hz, 1H, Ar-H), 7.56 (t, *J* = 7.6 Hz, 1H, Ar-H), 7.65 (t, *J* =7.6 Hz, 1H, Ar-H), 7.69 (d, *J* = 8.5 Hz, 1H, Ar-H), 7.72 (d, *J* = 7.6 Hz, 1H, Ar-H), 7.93 (d, *J* = 7.6 Hz, 1H, Ar-H). ^13^C NMR (213MHz, DMSO-d_6_), δ (ppm): 36.96 (-O-CH_2-_), Ar-C (111.71, 121.27, 122.21, 123.18, 123.84, 126.87, 127.99, 131.19, 131.37, 131.83, 133.53, 136.22, 162.05 (oxadiazole, C=N), 162.89 (oxadiazole, C=N), 169.08 (benzothiazole, C=N). ESI +ve MS (m/z): (100%) 344 [M + H]^+^, (35%) 346 [M + 2 + H]^+^. Anal. Calc. for C_16_H_10_ClO_2_N_3_S: C, 55.90; H, 2.93; O, 9.31; N, 12.22; S, 9.33. Found: C, 55.88; H, 2.94; O, 9.32; N, 12.21; S, 9.32.

#### 2.2.8. 2-((5-(4-Bromophenyl)-1,3,4-oxadiazol-2-yl)methoxy)benzo[d]thiazole (6)

Yield 63%, white, mp. 219–220 °C, M.W. 387, R_f_ = 0.54 in petroleum ether/ethylacetate (6 : 4) as a developing solvent. IR (ATR, cm^-1^), 3035 (C-H aromatic), 2869 (C-H aliphatic), 1677 (C=N of benzothiazole), 1590 (C=N of oxadiazole), 1495, 1473, 1331, 1187, 1069, 1023, 1010, 744, 716 (C-S). ^1^H NMR (850 MHz, DMSO-d_6_), δ (ppm): 5.56 (s, 2H, -OCH_2-_), 7.03 (d, *J* = 8.5 Hz, 2H, Ar-H), 7.45 (d, *J* = 7.65 Hz, 1H, Ar-H), 7.82 - 7.87 (m, 4H, Ar-H), 8.06 (d, *J* = 8.5 Hz, 1H. Ar-H). ^13^C NMR (213 MHz, DMSO-d_6_), δ (ppm): 43.24 (-O-CH_2-_), Ar-C (111.52, 121.17, 122.88, 123.39, 125.79, 127.60, 128.55, 129.77, 131.33, 131.63, 131.72, 137.10), 164.66 (oxadiazole, C=N), 164.69 (oxadiazole, C=N), 169.18 (benzothiazole, C=N). ESI +ve MS (m/z): 388 [M + H]^+^, 390 [M + 2 + H]^+^. Anal. Calc. for C_16_H_10_BrO_2_N_3_S: C, 49.50; H, 2.60; O, 8.24; N, 10.82; S, 8.26. Found: C, 49.52; H, 2.60; O, 8.26; N, 10.80; S, 8.23.

#### 2.2.9. 2-(5-((Benzo[d]thiazol-2-yloxy)-methyl)-1,3,4-oxadiazol-2-yl) phenol (7)

Yield 63%, white, mp. 142–143 °C, M.W. 325, R_f_ = 0.48 in petroleum ether/ethylacetate (6 : 4) as a developing solvent. IR (ATR, cm^-1^): 3261 (Ar-OH), 3070 (C-H aromatic), 2987 (C-H aliphatic), 1779 (C=N of benzothiazole), 1541 (C=N of oxadiazole), 1507, 1473, 1288, 1245, 1193, 1156, 1050, 745 (C-S). ^1^H NMR (850 MHz, DMSO-d_6_), δ (ppm): 5.64 (s, 2H, -O-CH_2-_), 7.27–7.31 (m, 1H, Ar-H), 7.42–7.44 (m, 1H, Ar-H), 7.52–7.54 (m, 3H, Ar-H), 7.70–7.74 (m, 1H, Ar-H), 7.79–7.83 (m, 2H, Ar-H), 10.51 (s, 1H, Ar-OH). ^13^C NMR (213 MHz, DMSO-d_6_), δ (ppm): 43.24 (-O-CH_2-_), Ar-C (111.68, 122.86, 123.18, 123.37, 123.81, 126.59, 126.87, 129.47, 132.21, 132.52, 136.27), 164.84 (oxadiazole, C=N), 165.63 (oxadiazole, C=N), 169.06 (benzothiazole, C=N). ESI +ve MS (m/z): 326 [M + H]^+^. Anal. Calc. for C_16_H_11_O_3_N_3_S: C, 59.07; H, 3.41; O, 14.75; N, 12.92; S, 9.86. Found: C, 59.09; H, 3.42; O, 14.77; N, 12.90; S, 9.85 (refer to supplementary materials).

#### 2.2.10. 3-(5-((Benzo[d]thiazol-2-yloxy)methyl)-1,3,4-oxadiazol-2-yl)benzenamine (8)

Yield: 55%, white, mp. 214–216 °C, M.W. 324, R_f_ = 0.50 in petroleum ether/ethylacetate (6 : 4) as a developing solvent. IR (ATR, cm^-1^): 3274 (NH2) , 3060 (C-H aromatic), 2988 (C-H aliphatic), 1651 (C=N of benzothiazole), 1587 (C=N of oxadiazole), 1473, 1434, 1329, 1196, 1052, 894, 742 (C-S). ^1^H NMR (850 MHz, DMSO-d_6_), δ (ppm): 4.77 (s, 2H, -O-CH_2-_), 6.76 (d, *J* = 6.8 Hz, Ar-H), 7.10–7.26 (m, 2H, Ar-H), 7.50–7.74 (m, 1H, Ar-H), 7.98–8.02 (m, 1H, Ar-H), 8.29–8.43 (m, 2H, Ar-H), 10.30 (s, 2H, Ar-NH2) . ^13^C NMR (213 MHz, DMSO-d_6_), δ (ppm): 43.27 (-O-CH_2-_), Ar-C (111.58, 121.17, 122.50, 123.39, 123.72, 126.60, 128.70, 128.97, 131.27, 132.92, 135.40, 135.65, 149.03), 165.53 (oxadiazole, C=N), 165.84 (oxadiazole, C=N), 169.24 (benzothiazole, C=N). ESI +ve MS (m/z): 325 [M + H]^+^. Anal. Calc. for C_16_H_12_O_2_N_4_S: C, 59.25; H, 3.73; O, 9.87; N, 17.27; S, 9.89. Found: C, 59.26; H, 3.74; O, 9.88; N, 17.25; S, 9.88.

#### 2.2.11. 2-((5-p-Tolyl-1,3,4-oxadiazol-2-yl)methoxy)benzo[d]thiazole (9)

Yield 68%, white, mp. 225–226 °C, M.W. 323, R_f_ = 0.66 in petroleum ether/ethylacetate (6 : 4) as a developing solvent. IR (ATR, cm^-1^): 3060 (C-H aromatic), 2984 (C-H aliphatic), 1651 (C=N of benzothiazole), 1593 (C=N of oxadiazole), 1489, 1473, 1192, 1042, 829, 746 (C-S). ^1^H NMR (850 MHz, DMSO-d_6_), δ (ppm): 2.35 (s, 3H, Ar-CH3) , 4.76 (s, 2H, -O-CH_2-_), 7.21–7.24 (m, 2H, Ar-H),7.29 (d, *J* =8.5 Hz, 2H, Ar-H), 7.37–7.41 (m, 1H, Ar-H), 7.66–7.68 (m, 1H, Ar-H), 7.76 (d, *J* =8.5 Hz, 2H, Ar-H). ^13^C NMR (213 MHz, DMSO-d_6_), δ (ppm): 21.04 (Ar-CH_3_), 43.24 (-O-CH_2-_), Ar-C (111.54, 121.16, 122.86, 123.37, 126.59, 127.67, 129.21, 130.09, 137.11, 142.0), 165.41 (oxadiazole, C=N), 165.58 (oxadiazole, C=N), 169.22 (benzothiazole, C=N); ESI +ve MS (m/z): 324 [M + H]^+^. Anal. Calc. for C_17_H_13_O_2_N_3_S: C, 63.14; H, 4.05; O, 9.90; N, 12.99; S, 9.92. Found: C, 63.12; H, 4.03; O, 9.91; N, 12.97; S, 9.91.

#### 2.2.12. 2-((5-m-Tolyl-1,3,4-oxadiazol-2-yl)methoxy)benzo[d]thiazole (10)

Yield 65%; white, mp. 179–180 °C, M.W. 323, R_f_ = 0.60 in petroleum ether/ethylacetate (6 : 4) as a developing solvent. IR (ATR, cm^-1^): 3039 (C-H aromatic), 2915 (C-H aliphatic), 1670 (C=N of benzothiazole), 1584 (C=N of oxadiazole), 1473, 1183, 1025, 761 (C-S). ^1^H NMR (850 MHz, DMSO-d_6_), δ (ppm): 2.41 (s, 3H, Ar-CH_3_), 4.81 (s, 2H, -O-CH_2_), 7.27–7.32 (m, 2H, Ar-H), 7.42–7.45 (m, 3H, Ar-H), 7.70–7.74 (m, 2H, Ar-H), 7.78–7.79 (m, 1H, Ar-H). ^13^C NMR (213 MHz, DMSO-d_6_), δ (ppm): 20.93 (Ar-CH3) , 43.24 (-O-CH_2-_), Ar-C (111.54, 121.16, 122.86, 123.18, 124.57, 126.59, 128.04, 128.59, 132.21, 132.52, 137.11, 137.85), 165.54 (oxadiazole, C=N), 165.63 (oxadiazole, C=N), 169.22 (benzothiazole, C=N). ESI +ve MS (m/z): 324 [M + H]^+^. Anal. Calc. for C_17_H_13_O_2_N_3_S: C, 63.14; H, 4.05; O, 9.90; N, 12.99; S, 9.92. Found: C, 63.12; H, 4.06; O, 9.92; N, 12.97; S, 9.91.

#### 2.2.13. 2-((5-((1H-indol-3-yl)methyl)-1,3,4-oxadiazol-2-yl)methoxy)benzo[d]thiazole (11)

Yield 56%, yellow, mp. 172–173 °C, M.W. 362, R_f_ = 0.52 in petroleum ether/ethylacetate (6 : 4) as a developing solvent. IR (ATR, cm^-1^): 3254 (C-H aromatic), 2987 (C-H aliphatic), 1669 (C=N of benzothiazole), 1592 (C=N of oxadiazole), 1540, 1473, 1242, 1177, 1044, 745 (C-S). ^1^H NMR (850 MHz, DMSO-d_6_),δ (ppm): 3.17 (s, 2H, -CH_2_), 4.86 (s, 2H, -O-CH_2-_), 6.97–7.73 (m, 9H, Ar-H), 11.21 (s, 1H, NH). ^13^C NMR (213 MHz, DMSO-d_6_), δ (ppm): 23.42 (-CH_2_), 43.44 (-O-CH_2-_), Ar-C (111.44, 121.86, 123.98, 124.38, 125.89, 126.45, 128.34, 128.59, 132.58, 132.64, 137.11, 137.75, 145.67), 165.44 (oxadiazole, C=N), 165.72 (oxadiazole, C=N), 169.68 (benzothiazole, C=N). ESI +ve MS (m/z): 363 [M + H]^+^. Anal. Calc. for C_19_H_14_O_2_N_4_S: C, 62.97; H, 3.89; O, 8.83; N, 15.46; S, 8.85. Found: C, 62.95; H, 3.90; O, 8.84; N, 15.46; S, 8.84.

#### 2.2.14. 2-((5-(3-Chlorophenyl)-1,3,4-oxadiazol-2-yl)methoxy)benzo[d]thiazole (12)

Yield 69%, white, mp. 235–236 °C, M.W. 343,R_f_ = 0.66 in petroleum ether/ethylacetate (6 : 4) as a developing solvent. IR (ATR, cm^-1^): 3037 (C-H aromatic), 2976 (C-H aliphatic), 1655 (C=N of benzothiazole), 1594 (C=N of oxadiazole), 1582, 1473, 1334, 1244, 1157, 1048, 744 (C-S). 1 H NMR (850 MHz, DMSO-d_6_6), δ (ppm): 4.77 (s, 2H, -O-CH_2-_), 7.21–7.25 (m, 2H, Ar-H), 7.37–7.39 (m, 1H, Ar-H), 7.54 (t, *J* =7.3 Hz, 1H, Ar-H), 7.64–7.68 (m, 2H, Ar-H), 7.82 (d, *J* = 8.5 Hz, 1H, Ar-H), 7.89 (t, *J* = 1.7 Hz, 1H, Ar-H). ^13^C NMR (213 MHz, DMSO-d_6_), δ (ppm): 43.25 (-O-CH_2_), Ar-C (111.52, 121.18, 122.89, 123.40, 126.22, 126.60, 127.27, 130.65, 131.84, 133.36, 137.10), 164.12 (oxadiazole, C=N), 165.50 (oxadiazole, C=N), 169.24 (benzothiazole, C=N). ESI +ve MS (m/z): (80%) 344 [M + H]^+^, (28%) 346 [M + 2 + H]^+^. Anal. Calc. for C_16_H_10_ClO_2_N_3_S: C, 55.90; H, 2.93; O, 9.31; N, 12.22; S, 9.33. Found: C, 55.88; H, 2.92; O, 9.32; N, 1.23; S, 9.32.

#### 2.2.15. 2-((5-(3-Nitrophenyl)-1,3,4-oxadiazol-2-yl)methoxy)benzo[d]thiazole (13)

Yield 57%, white, mp. 180–181 °C, M.W. 354, R_f_ = 0.54 in petroleum ether/ethylacetate (6 : 4) as a developing solvent. IR (ATR, cm^-1^): 3038 (C-H aromatic), 2986 (C-H aliphatic), 1673 (C=N of benzothiazole), 1605, 1527 (C=N of oxadiazole), 1473, 1343 (NO_2_), 1286, 1189, 1024, 746 (C-S). ^1^H NMR (850 MHz, DMSOd_6_), δ (ppm): 4.79 (s, 2H, -O-CH_2-_), 7.22–7.26 (m, 2H, Ar-H), 7.38–7.40 (m, 1H, Ar-H), 7.68 (dd, *J* = 0.85, 7.6 Hz, 1H, Ar-H), 7.82 (t, *J* = 7.6, 1H, Ar-H), 8.30 (d, *J* = 8.5, 1H, Ar-H), 8.42 - 8.44 (m, 1H, Ar-H), 8.69 (t, *J* = 1.7 Hz, 1H, Ar-H). ^13^C NMR (213 MHz, DMSO-d_6_), δ (ppm): 43.25 (-O-CH_2_), Ar-C (111.50, 121.19, 122.25, 122.91, 123.41, 126.62, 130.50, 133.55, 137.10, 147.85), 163.48 (oxadiazole, C=N), 165.53 (oxadiazole, C=N), 169.25 (benzothiazole, C=N). ESI +ve MS (m/z): 355 [M + H]^+^. Anal. Calc. for C_16_H_10_O_4_N_4_S: C, 54.23; H, 2.84; O, 18.06, N, 15.81; S, 9.05. Found: C, 54.25; H, 2.84; O, 18.07; N, 15.80; S, 9.05.

#### 2.2.16. 2-(5-((Benzo[d]thiazol-2-yloxy)methyl)-1,3,4-oxadiazol-2-yl)benzenethiol (14)

Yield 56%, white, mp. 243–244 °C, M.W. 341, R_f_ = 0.58 in petroleum ether/ethylacetate (6 : 4) as a developing solvent. IR (ATR, cm^-1^): 3050 (C-H aromatic), 2988 (C-H aliphatic), 2567 (Ar-SH), 1668 (C=N of benzothiazole), 1587 (C=N of oxadiazole), 1473, 1269, 1197, 897, 741 (C-S). ^1^H NMR (850 MHz, DMSO-d_6_), δ (ppm): 3.17 (s, 1H, Ar-SH), 4.80 (s, 2H, -O-CH_2-_), 7.38–7.50 (m, 4H, Ar-H), 7.61–7.79 (m, 2H, Ar-H), 8.01–8.05 (m, 1H, Ar-H), 8.23–8.24 (m, 1H, Ar-H). ^13^C NMR (213 MHz, DMSO-d_6_), δ (ppm): 43.175 (-O-CH_2-_), Ar-C (111.31, 123.68, 123.86, 124.04, 126.32, 128.73, 131.80, 132.0, 135.69, 137.49, 140.69, 141.72), 161.72 (oxadiazole, C=N), 161.99 (oxadiazole, C=N), 167.14 (benzothiazole, C=N). ESI +ve MS (m/z): 342 [M + H]^+^. Anal. Calc. for C_16_H_11_O_2_N_3_S_2_: C, 56.29; H, 3.25; O, 9.37; N, 12.31; S, 18.78. Found: C, 56.27; H, 3.26; O, 9.38; N, 12.30; S, 18.79.

#### 2.2.17. 2-((5-o-Tolyl-1,3,4-oxadiazol-2-yl)methoxy)benzo[d]thiazole (15)

Yield 63%, white, mp. 282–283 °C, M.W. 323, R_f_ = 0.62 in petroleum ether/ethylacetate (6 : 4) as a developing solvent. IR (ATR, cm^-1^): 3060 (C-H aromatic), 2987 (C-H aliphatic), 1670 (C=N of benzothiazole), 1592 (C=N of oxadiazole), 1489, 1473, 1176, 1044, 746 (C-S). ^1^H NMR (850 MHz, DMSO-d_6_), δ (ppm): 2.41 (s, 3H, Ar-CH_3_), 4.80 (s, 2H, -O-CH_2-_), 7.27–7.32 (m, 4H, Ar-H),7.40–7.49 (m, 2H, Ar-H), 7.54–7.57 (m, 1H, Ar-H), 7.77 (dd, *J* = 0.85 Hz, 7.6 Hz, 1H, Ar-H). ^13^C NMR (213 MHz, DMSO-d_6_), δ (ppm): 21.24 (Ar-CH_3_), 43.21 (-O-CH_2-_), Ar-C (111.71, 121.27, 122.19, 123.17, 123.81, 126.86, 127.36, 130.88, 131.74, 131.83, 134.59, 136.26), 164.94 (oxadiazole, C=N), 165.45 (oxadiazole, C=N), 169.07 (benzothiazole, C=N). ESI +ve MS (m/z): 324 [M + H]^+^. Anal. Calc. for C_17_H_13_O_2_N_3_S: C, 63.14; H, 4.05; O, 9.90; N, 12.99; S, 9.92. Found: C, 63.11; H, 4.05; O, 9.91; N, 13.00; S, 9.91.

#### 2.2.18. 2-((5-(4-Nitrophenyl)-1,3,4-oxadiazol-2-yl)methoxy)benzo[d]thiazole (16)

Yield 63%, white, mp. 223–225 °C, M.W. 354, R_f_ = 0.56 in petroleum ether/ethylacetate (6 : 4) as a developing solvent. IR (ATR, cm^-1^): 3035 (C-H aromatic), 2981 (C-H aliphatic), 1667 (C=N of benzothiazole), 1522 (C=N of oxadiazole), 1472, 1335 (NO_2_), 1249, 1022, 747 (C-S). ^1^H NMR (850 MHz, DMSO-d_6_), δ (ppm): 4.79 (s, 2H, -O-CH_2-_), 7.22–7.26 (m, 2H, Ar-H), 7.38 (t, *J* = 7.6, 1H, Ar-H), 7.68 (d, *J* = 7.6, 1H, Ar-H), 8.08 (d, *J* = 8.2, 2H, Ar-H), 8.34 (d, *J* = 8.5, 2H, Ar-H). ^13^C NMR (213 MHz, DMSO-d_6_), δ (ppm): 43.24 (-O-CH_2-_), Ar-C (111.50, 121.18, 122.90, 123.40, 123.78, 126.60, 129.04, 137.09, 137.83, 149.46), 163.96 (oxadiazole, C=N), 165.51 (oxadiazole, C=N), 169.24 (benzothiazole, C=N). ESI +ve MS (m/z): 355 [M + H]^+^. Anal. Calc. for C_16_H_10_O_4_N_4_S: C, 54.23; H, 2.84; O, 18.06; N, 15.81; S, 9.05. Found: C, 54.20; H, 2.84; O, 18.07; N, 15.82; S, 9.05.

#### 2.2.19. 2-((5-((2,5-Dichlorophenoxy)methyl)-1,3,4-oxadiazol-2-yl)methoxy)benzo [d] thiazole (17)

Yield 54%, white, mp. 243–244 °C, M.W. 407, R_f_ = 0.50 in petroleum ether/ethylacetate (6 : 4) as a developing solvent. IR (ATR, cm^-1^): 3070 (C-H aromatic), 2988 (C-H aliphatic), 1668 (C=N of benzothiazole), 1586 (C=N of oxadiazole), 1473, 1460, 1269, 1234, 1187, 1093, 1052, 898, 739 (C-S). ^1^H NMR (850 MHz, DMSO-d_6_), δ (ppm): 4.71 (s, 2H, -O-CH_2-_), 4.74 (s, 2H, -O-CH_2-_), 7.07 (d, *J* = 9.5 Hz, 1H, Ar-H), 7.16–7.22 (m, 2H, Ar-H), 7.27–7.37 (m, 2H, Ar-H), 7.49 (s, 1H, Ar-H), 7.56–7.58 (m, 1H, Ar-H). ^13^C NMR (213 MHz, DMSO-d_6_), δ (ppm): 43.55 (-O-CH_2-_), 43.74 (-O-CH_2-_), Ar-C (111.88, 121.58, 123.00, 123.31, 123.81, 127.03, 128.07, 128.44, 129.43, 129.85, 130.02, 137.51), 165.47 (oxadiazole, C=N), 165.87 (oxadiazole, C=N), 169.62 (benzothiazole, C=N). ESI +ve MS (m/z): (56%) 408 [M + H]^+^, (39%) 410 [M + 2 + H]^+^. Anal. Calc. for C_17_H_11_Cl_2_O_3_N_3_S: C, 50.01; H, 2.72; O, 11.76; N, 10.29; S, 7.85. Found: C, 49.98; H, 2.71; O, 11.77; N, 10.30; S,7.85.

#### 2.2.20. 2-((5-Styryl-1,3,4-oxadiazol-2-yl)methoxy)benzo[d]thiazole (18)

Yield 58%, white, mp. 192–193 °C, M.W. 335, R_f_ = 0.52 in petroleum ether/ethylacetate (6 : 4) as a developing solvent. IR (ATR, cm^-1^): 3067 (C-H aromatic), 2983 (C-H aliphatic), 1600 (C=N of benzothiazole), 1577 (C=N of oxadiazole), 1538, 1508, 1484, 1449, 1403, 1312, 1289, 1217, 1160, 1089, 1047, 744 (C-S). ^1^H NMR (850 MHz, DMSO-d_6_), δ (ppm): 5.55 (s, 2H, -O-CH_2-_), 7.25–7.27 (m, 1H, Ar-H), 7.29–7.33 (m, 1H, Ar-H), 7.40–7.45 (m, 5H, Ar-H), 7.53–7.57 (m, 1H, Ar-H), 7.72–7.73 (m, 1H, Ar-H), 7.76 (d, *J* = 6.8, 2H, -C=CH). ^13^C NMR (213 MHz, DMSO-d_6_), δ (ppm): 37.02 (-O-CH_2-_), Ar-C (109.75, 111.66, 121.26, 123.18, 123.81, 126.88, 127.96, 128.99, 130.13, 134.47, 136.27), 139.28 (C=C), 160.91 (oxadiazole, C=N), 164.75 (oxadiazole, C=N), 169.05 (benzothiazole, C=N). ESI +ve MS (m/z): 336 [M + H]^+^. Anal. Calc. for C_18_H_13_O_2_N_3_S: C, 64.46; H, 3.91; O, 9.54; N, 12.53; S, 9.56. Found: C, 64.44; H, 3.90; O, 9.55; N,12.54; S, 9.57.

### 2.3. Antibacterial activity

#### 2.3.1. In vitro susceptibility test

The antibacterial study of the synthesized compounds was carried out against the following human pathogenic bacterial strains: *Pseudomonas aeruginosa* (ATCC 27853); *Staphylococcus aureus* (ATCC 25923); *Escherichia coli* (ATCC 25922); *Proteus mirabilis* (ATCC 13376); *Staphylococcus epidermidis* (ATCC 12228); *Enterococcus faecalis* (ATCC 29212).The study was performed at Albaha Regional Research Laboratory, Albaha, Kingdom of Saudi Arabia. The susceptibility test was performed using the disc diffusion method [20]. The tested compounds were dissolved in DMF to prepare a chemical stock solution of 2 mg/mL. Each bacterial strain was suspended in Mueller Hinton (MH) broth and diluted to an approximately 106 colony-forming unit (cfu/mL); they were floodinoculated onto the surface of MH agar and Sabouraud dextrose agar (SDA) and then dried. For *Pseudomonas aeruginosa* , Macconcy agar was used; for *Escherichia coli* and *Staphylococcus aureus* , Mueller Hinton agar was used. Six-millimeter diameter discs were prepared, and 200 μg of each compound was loaded onto the discs. Antibacterial activity was evaluated by measuring the zone of inhibition against the tested bacterial strains. Amoxicillin was used as a standard drug. DMF was used as a solvent (negative controls).

#### 2.3.2. Minimum inhibitory concentration

The minimum inhibitory concentration (MIC) was determined by the conventional paper disc diffusion method. The compounds showing a promising zone of inhibition were dissolved in DMF and loaded on the disks by micropipette at different concentrations (100, 50, 25, 12.50, 6.25, 3.125, and 1.56 μg/disc). The loaded discs were kept on a microbe-inoculated agar plate surface. The plates were kept at 37 °C for 24 h; each experiment was repeated 3 times, and MIC was expressed as the lowest concentration at which inhibition of the test organism took place.

## 3. Results and discussion

**Figure 2:**
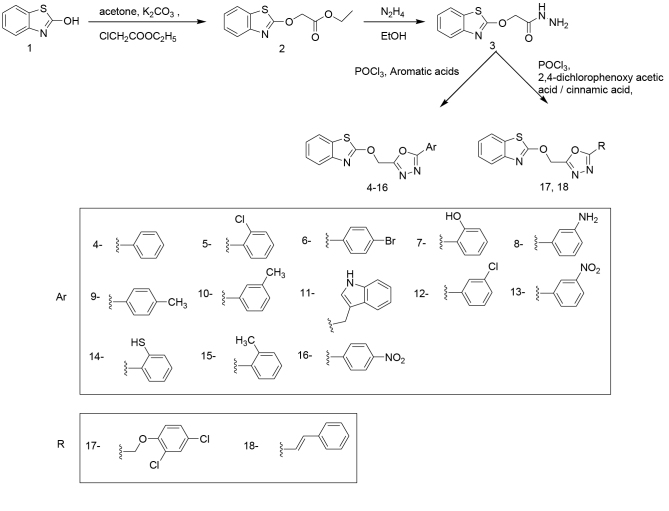
A synthesis scheme of 2-hydroxy benzothiazole based 1,3,4-oxadiazole derivatives.

**Figure 3:**
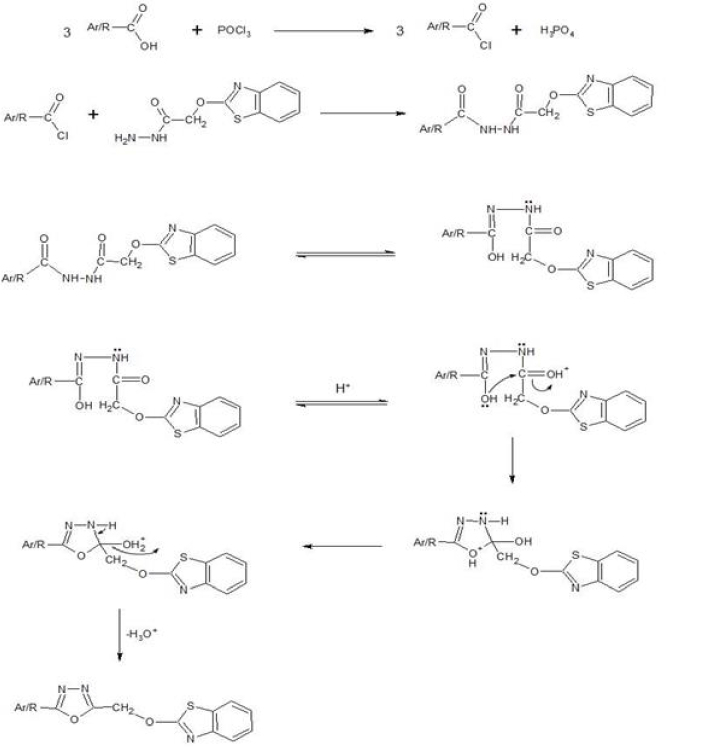
Mechanism for the formation of 1,3,4-oxadiazole derivatives.

A series of 15 new 2-hydroxy benzothiazole linked 1,3,4-oxadiazole derivatives ( **4-18** ) have been synthesized according to the route described in Scheme 1 (Figure 2). The reaction of 2-hydroxy benzothiazole ( **1** ) with ethyl chloroacetate in the presence of anhydrous potassium carbonate and dry acetone yielded ethyl 2-(benzo[d]thiazol-2-yloxy)acetate ( **2** ), which, on further reaction with hydrazine monohydrate in absolute ethanol, yielded a key intermediate 2-(benzo[d]thiazol-2-yloxy)acetohydrazide ( **3** ). This key intermediate ( **3** ) reacted with different aromatic acids, substituted phenoxy acetic acids, and cinnamic acid in the presence of dehydrating agent POCl3 to give the target compounds ( **4-18** ). The mechanism for the formation of 1,3,4-oxadiazole derivatives is shown in Figure 3. The proposed structures of the synthesized compounds were confirmed with different analytical techniques, such as IR, ^1^H NMR, and ^13^C NMR spectroscopy, an elemental analyzer, and mass spectrometry. All of the spectral data were in agreement with the proposed structures of the synthesized compounds.

Formation of ethyl 2-(benzo[d]thiazol-2-yloxy) acetate ( **2** ) was confirmed by the presence of a strong absorption band at 1671 cm^-1^ for C=N of the benzothiazole ring, 1663 cm^-1^ for carbonyl carbon, and 1049 cm^-1^ for C-O of the ester group. The ^1^H NMR of intermediate (2) showed a quartet of 2 protons at δ 4.17 ppm ( *J* J =6.8 Hz) and a triplet of 3 protons at δ 1.21 ppm ( *J* =6.8 Hz), which is typical for ethyl ester. The methylene protons were observed as a singlet at δ 4.84 ppm in ^1^H NMR and at δ 61.46 ppm in ^13^C NMR. Furthermore, the aromatic protons of the benzothiazole ring appeared as a triplet of triplet integrating for 1 proton each at 7.23 ppm ( *J* =0.85 Hz, 8.5 Hz) and 7.36 ppm ( *J* =0.85 Hz, 8.5 Hz), 1 doublet at 7.31 ppm ( *J* =8.5 Hz) for 1 proton, and a doublet of doublet integrating for 1 proton at 7.68 ppm ( *J* = 0.85 Hz, 7.6 Hz). Finally, formation of compound ( **2** ) was confirmed by the appearance of a molecular ion peak at 238 (M + H)^+^ in the mass spectrum. Formation of acetohydrazide ( **3** ) was supported by the presence of absorption bands at 3311 cm^-1^ and 3222 cm^-1^ for NH_2_ and NH, respectively, and a carbonyl carbon stretching at 1653 cm^-1^ in the IR spectrum. Substitution of the ethoxy group by hydrazidic groups was supported by the disappearance of signals of ethyl protons of the ester group and the appearance of hydrazidic protons as a broad singlet at δ 9.45 ppm for 1 proton (-NH), and another broad singlet at δ 4.31 ppm for 2 protons (NH_2_) in the ^1^H NMR spectrum. The structure of compound ( **3** ) was confirmed by mass spectrometry, which showed a molecular ion peak at 224 (M + H)^+^. The formation of target compounds ( **4-18** ) was confirmed by the disappearance of absorption bands of NH-NH_2_ and the carbonyl group, and the appearance of an absorption band of the oxadiazole ring at 1522–1594 cm^-1^ for C=N stretching in the IR spectra, indicating the formation of an oxadiazole ring. Further structural confirmation of the target compounds **4-18** was made using ^1^H NMR and ^13^C NMR spectroscopy. Disappearance of hydrazidic protons as a broad singlet at δ 9.45 ppm for 1 proton (-NH) and another broad singlet at δ 4.31 ppm for 2 protons (NH_2_) and the appearance of extra aromatic protons in ^1^H NMR spectra of **4-18** . The appearance of 2 additional signals in the range δ 160–165 ppm for C=N of the oxadiazole ring and 1 signal in the range δ 167–169 ppm for C=N of the benzothiazole ring in the ^13^C NMR spectra supported the formation of **4-18** . All of the target compounds were confirmed from the mass spectral data.

The capacity of a drug to exhibit a pharmacological or therapeutic effect is related to the influence of various physicochemical properties of the drug on the biomolecule that it interacts with. In silico approaches are being used today in drug discovery to assess the ADME (absorption, distribution, metabolism, excretion) properties of compounds at the early stages of discovery to generate potential lead molecules. Different physiochemical parameters of drug candidates play a crucial role in their pharmacokinetic behavior [21]. In view of this, their calculation and measurement aids in prioritizing compounds for screening as efficient drug candidates and prevents premature decisions in drug discovery [22]. A molecule likely to be developed as an orally active drug should obey the Lipinski rule of 5 [23], which states the following 4 criteria: partition coefficient (Clog P) ≤5, molecular weight (MW) ≤500, number of hydrogen bond acceptors ≤10. (HBA), and number of hydrogen bond donors ≤5 (HBD). Violation of more than one of these rules would result in problems in bioavailability upon oral administration. According to Veber et al. [24], the number of rotatable bonds should be ≤10, which is an indicator for good bioavailability. In the present study, we calculated several parameters for predicting drug likeness properties of synthesized compounds in order to screen potential candidate drugs. The prepared 2-hydroxy benzothiazole linked 1, 3, 4-oxadiazole conjugates ( **4-18** ) were subjected to in silico physicochemical studies such as number of rotatable bonds (nROTB), hydrogen bond acceptor (HBA), hydrogen bond donor (HBD), lipophilicity (iLogP), and topological polar surface area (TPSA), which were calculated in order to understand the drug’s transport properties. In silico percentage absorption was calculated using the reported formula [(%ABS = 109 – (0.345 ×TPSA)] [25]. As observed from Table 1, absorption was in the range of 61.71%–86.77%; compound 4 showed the highest in silico percentage absorption (86.77%), whereas most of the compounds showed more than 70% absorption. All of the synthesized compounds **4-18** followed the Lipinski rule of 5. MW range was found to be in the range 310–410 (<500), HBA range 3–7 (≤10), HBD range 0–1 (≤5), and iLogP (lipophilicity) range between 2.69–3.61 (≤5), suggesting that the compounds upon administration possess good drug likeness properties (Tables 2 and 3). Furthermore, all of the compounds comply with the Veber rule, wherein nROTB was found to be in the range 5–7 (<10), suggesting good bioavailability. All of the tested compounds revealed a TPSA range of 63.5–135.1 Å^2^(<140 Å^2^), which indicates good intestinal absorption. It was also interesting that only compound 4 was able to permeate through the blood brain barrier (BBB). Also of note is that all of the tested compounds were CYP1A2, CYP2C19, and CYP2C9 inhibitors.

**Table 1:** Physicochemical properties of the synthesized compounds 4–18.

Compd no.	nROTB^a^	HBA^b^	HBD^c^	iLogP^d^	LogS^e^	TPSA^f^	In silico % absorption
4	4	3	0	3.59	MS	63.5	86.77
5	4	5	0	3.2	MS	89.28	77.75
6	4	5	0	3.44	MS	89.28	77.75
7	4	6	1	2.97	MS	109.51	70.67
8	4	5	1	2.69	MS	115.3	68.64
9	4	5	0	3.31	MS	89.28	77.75
10	4	5	0	3.44	MS	89.28	77.75
11	5	5	1	2.85	MS	105.07	72.22
12	4	5	0	3.59	MS	89.28	77.75
13	5	7	0	2.96	MS	135.1	61.71
14	4	5	0	3.23	MS	128.08	64.17
15	4	5	0	3.3	MS	89.28	77.75
16	5	7	0	2.96	MS	135.1	61.71
17	6	6	0	3.61	MS	98.51	74.52
18	5	5	0	3.4	MS	89.28	77.75

^a^nROTB: Number of rotable bonds; ^b^HBA: Number of hydrogen bond acceptor; ^c^HDC: Number of hydrogen bond donor; ^d^iLogP: Lipophilicity; ^e^LogS: Water solubility (MS: Moderately soluble, PS: Poorly soluble); ^f^TPSA: Topological polar surface area (0A2).

**Table 2:** Pharmacokinetic/ADME predictions of the synthesized compounds **4-18** .

Compd.	Pharmacokinetic/ADME properties							
	GI Abs^a^	BBB^b^	CYP1A2 inhibitor^d^	CYP2C19 inhibitor^e^	CYP2C9 inhibitor^f^	CYP2D6 inhibitor^g^	CYP3A4 inhibitor^h^	LogKp^i^
4	High	Yes	Yes	Yes	Yes	Yes	Yes	-4.67
5	High	No	Yes	Yes	Yes	No	No	-5.32
6	High	No	Yes	Yes	Yes	No	No	-5.55
7	High	No	Yes	Yes	Yes	Yes	Yes	-5.91
8	High	No	Yes	Yes	Yes	Yes	Yes	-6.13
9	High	No	Yes	Yes	Yes	No	Yes	–5.38
10	High	No	Yes	Yes	Yes	No	Yes	–5.38
11	High	No	Yes	Yes	Yes	Yes	Yes	–5.58
12	High	No	Yes	Yes	Yes	No	No	–5.32
13	Low	No	Yes	Yes	Yes	No	No	–5.96
14	Low	No	Yes	Yes	Yes	No	Yes	–5.65
15	High	No	Yes	Yes	Yes	No	Yes	–5.38
16	Low	No	Yes	Yes	Yes	No	No	–5.96
17	High	No	Yes	Yes	Yes	No	Yes	–5.38
18	High	No	Yes	Yes	Yes	No	No	–5.26

^a^GI Abs: Gastro intestinal absorption; ^b^BBB: Blood brain barrier permeant; ^d^CYP1A2: Cytochrome P450 family1 subfamily A member 2 (PDBHI4); ^e^CYP2C19: Cytochrome P450 family2 subfamily C member 19 (PDB4GQS); ^f^CYP2C9: Cytochrome P450 family2 subfamily C member 9 (PDB1OG2); ^g^CYP2D6: Cytochrome P450 family 2 subfamily D member 6 (PDB5TFT); ^h^CYP3A4: Cytochrome P450 family 3 subfamily A member 4 (PDB4K9T); ^i^Skin permeation in cm/s.

**Table 3:** Drug likeness predictions of the synthesized compound **4-18** .

Compd. no.	Lipinski violation	Ghose violation	Veber violation	Egan violation	Muegge violation	Bioavailability score
4	0	0	0	0	0	0.55
5	0	0	0	0	0	0.55
6	0	0	0	0	0	0.55
7	0	0	0	0	0	0.55
8	0	0	0	0	0	0.55
9	0	0	0	0	0	0.55
10	0	0	0	0	0	0.55
11	0	0	0	0	0	0.55
12	0	0	0	0	0	0.55
13	0	0	0	1	0	0.55
14	0	0	0	0	0	0.55
15	0	0	0	0	0	0.55
16	0	0	0	1	0	0.55
17	0	0	0	0	0	0.55
18	0	0	0	0	0	0.55

The newly synthesized compounds were evaluated for in vitro antibacterial activity against Gram-positive bacteria ( *S. epidermidis* , *S. aureus* , and *E. faecalis* ) and Gram-negative bacteria ( *E. coli* , *P. mirabilis* , and *P. aerogenosa* ). From the results of Table 4, synthesized **4-18** compounds showed excellent to moderate activity. Compounds **5** , **9** , **12** , **14** , **15** , **16** , and **17** were the most active compounds against the tested bacterial strains. Compound **5** showed a zone of inhibition of 20 mm against *S. epidermidis* , *E. faecalis* , and *E. coli* , which was comparable to the positive control amoxicillin, which had zones of inhibition of 26 mm, 22 mm, and 20 mm, respectively. Compound **8** was found to be superior to amoxicillin, having a zone of inhibition of 14 ±1.3 mm against *P. aeruginosa* . Compound **17** exhibited comparable zones of inhibition (24 ±1.7 mm, 23 ±1.5 mm, 20 ±1.3 mm, 18 ±1.4 mm, 10 ±1.1 mm) with the standard drug amoxicillin, having zones of inhibition of 26 ±1.6 mm, 25 ±1.4 mm, 22 ±1.6 mm, 20 ±1.4 mm, and 12 ±1.2 mm against *S. epidermidis* , *S. aureus* , *E. faecalis* , *E. coli* , and *P. aeruginosa* , respectively. Compound 14 was active against *S. epidermidis* , *S. aureus* , *E. faecalis* , and *P. aeruginosa* and resistant toward *E. coli* and *P. mirabilis* . Compound **15** revealed zones of inhibition of 22 ±1.6 mm, 23 ±1.4 mm, and 20 ±1.4 mm against *S.e* , *S.a* , and *E.c* , respectively. Compounds **5** , **7** , **14** , and **15** showed significant zones of inhibition (12 mm) compared with the standard drug amoxicillin. Compound **18** was found to be resistant to all of the bacterial strains except *S. aureus* . Compounds **10** , **11** , **12** , and **13** were resistant to *P. aeruginosa* .

**Table 4:** Antibacterial activity of the synthesized compounds 4–18 showing zone of inhibition in the form of mean ±SD (mm).

Antibacterial activity 200 μg/disc						
Compd. no.	Gram positive bacteria			Gram negative bacteria		
	*S.e*	*S.a*	*E.f*	*E.c*	*P.m*	*P.a*
4	-	-	-	-	-	-
**5**	20 ±1.4	11 ±1.3	20 ±1.2	20 ±1.6	12 ±1.2	12 ±1.4
6	9 ±1.0	10 ±1.1	10 ±1.1	14 ±1.5	-	-
7	12 ±1.2	10 ±1.2	8 ±1.0	12 ±1.2	-	-
8	17 ±1.4	14 ±1.3	15 ±1.2	12 ±1.2	10 ±1.1	14 ±1.3
**9**	20 ±1.3	15 ±1.2	16 ±1.3	14 ±1.2	10 ±1.1	12 ±1.2
10	18 ±1.2	16 ±1.3	12 ±1.1	14 ±1.2	10 ±1.1	-
11	17 ±1.3	16 ±1.2	14 ±1.2	16 ±1.3	-	-
**12**	20 ±1.3	14 ±1.3	12 ±1.1	12 ±1.2	-	-
13	17 ±1.2	12 ±1.1	11 ±1.1	15 ±1.2	-	-
**14**	24 ±1.4	22 ±1.3	20 ±1.4	16 ±1.3	10 ±1.1	12 ±1.1
**15**	22 ±1.6	23 ±1.4	18 ±1.3	20 ±1.4	11 ±1.1	12 ±1.3
**16**	20 ±1.6	18 ±1.3	-	10 ±1.4	-	-
**17**	24 ±1.7	23 ±1.5	20 ±1.3	18 ±1.4	8 ±0.8	10 ±1.1
18	-	08 ±0.7	-	-	-	-
Amoxicillin	26 ±1.6	25 ±1.4	22 ±1.6	20 ±1.4	25 ±1.8	12 ±1.2

S.e: Staphylococcus Epidermidis (ATCC 12228); S.a: Staphylococcus aureus (ATCC 25923); E.f: Enterococcus Faecalis (ATCC 29212); E.c: Escherichia Coli (ATCC25922); P.m: Proteus Merabilis (ATCC 13376); P.a: Pseudomonas Aerugenosa (ATCC 27853); —: No zone of inhibition.

The antibacterial activity of the most active compounds was further tested to determine minimum inhibitory concentration. It can be seen from Table 5 that compound **14** showed an MIC of 6.25 ±0.2 μg/disc comparable to that of the standard drug amoxicillin against all of the Gram-positive bacteria. Compounds **5** , **14** , and **17** exhibited MICs of 12.5 ±0.8 μg/disc, which was also comparable to the standard drug against *E. faecalis* ; compounds **5** , **15** , **17** showed the same MIC of 12.5 ±0.4 μg/disc against *E.coli* . From the above results, structure activity relationships (SAR) can be generated as follows: compound 17, possessing 2 chloro substituents at ortho and para positions on the phenyl ring, showed comparable activity to the standard drug amoxicillin, while mono chloro substituted phenyl rings such as compounds **5** (2-Cl) and **12** (3-Cl) resulted in diminishing activity. The antibacterial activity was found to be dependent on the electronegativity of the halogens. Compounds **5** , **12** , and **17** , having more electronegative chloro atoms on the ring, were found to be more promising in exerting antibacterial activity than compound **6** , which has less electronegative halogen such as the bromo group in the ring. Compound **5** having 2-Cl and **12** having 3-Cl showed almost comparable activity, which suggests that the activity is independent of the position of halogens on the ring. Compound **8** having an electron donating group (3-NH_2_) on the aromatic ring exhibited superior activity against *P. aeruginosa* . The presence of electron withdrawing groups such as NO_2_ also affected antibacterial activity. Compound **16** (NO_2_ group at para position) exhibited more promising antibacterial activity than compound **13** (NO_2_ group at meta position). This trend in activity may be due to a negative mesomeric effect of the NO_2_ group at the para position which is absent in the meta position. Compound **14** , having a thiol group, showed significant activity. The presence of a linker such as CH=CH (compound **18** ) and heterocyclic ring indole (compound **11** ) in the target compounds resulted in a loss in activity. The presence of an electron-donating methyl group on the phenyl ring showed excellent to moderate activity; the pattern was observed as compounds 15 >9 >10 for 2-Me, 4-Me, and 3-Me respectively. This trend in activity may be attributed to the hyperconjugation effect of the methyl group at ortho and para positions in compounds **15** and **9** , which is absent at the meta position in compound **10** . Compound **1** , which does not have any substituents on the phenyl ring, was found to be resistant against all tested bacterial strains. From these findings, it is clear that the presence of substituents on the phenyl ring is required for exerting antibacterial activity.

**Table 5:** Minimal inhibitory concentrations (MIC) of the active synthesized compounds against pathogenic bacteria tested^a^.

Compound	Gram positive bacteria			Gram negative bacteria
	*S.e*	*S.a*	*E.f*	*E.c*
5	12.5 ±0.5	100 ±0.8	12.5 ±0.2	12.5 ±0.3
9	12.5 ±0.4	25 ±0.9	25 ±0.5	50 ±0.4
12	12.5 ±0.7	25 ±0.3	50 ±0.9	50 ±0.8
14	6.25 ±0.6	3.12 ±0.4	12.5 ±0.3	25 ±0.6
15	12.5 ±0.4	6.25 ±0.8	25 ±0.4	12.5 ±0.7
16	12.5 ±0.8	6.25 ±0.7	>100	50 ±0.9
17	12.5 ±0.9	6.25 ±0.9	12.5 ±0.8	12.5 ±0.5
Amoxicillin	6.25 ±0.2	3.12 ±0.6	12.5 ±0.8	12.5 ±0.4

^a^Antibacterial activity were expressed as MIC in μg/disc.

In conclusion, a library of 15 2-hydroxy benzothiazole-linked 1,3,4-oxadiazole conjugates have been synthesized and confirmed by different analytical techniques. From the ADME predictions, compound **4** showed the highest in silico percentage absorption, 86.77%, while most of the compounds showed more than 70% absorption. It was observed that all of the compounds comply with the Lipinski rule of 5, which suggests that the compounds possess good drug likeness properties upon administration. Furthermore, all of the compounds follow the Veber rule, wherein nROTB was found to be in the range 5–7 (<10) and topological polar surface area in the range 63.5–135.1 Å^2^(<140 Å^2^), indicating good bioavailability and good intestinal absorption. From the antibacterial results, Compound 14 showed a comparable MIC (6.25 ±0.2 μg/disc) to that of the standard drug amoxicillin against all of the Gram-positive bacteria. Compounds **5** , **14** , and **17** exhibited an MIC of 12.5 ±0.8 μg/disc, which was comparable to that of the standard drug against *E. faecalis* , whereas compounds **5** , **15** , and **17** showed the same MIC (12.5 ±0.4 μg/disc) against *E. coli* . From these results, we conclude that these synthesized compounds may be used for the development of effective and safer antibacterial agents.

Supplementary Materials
