# Supplementary material for: In silico ADME predictions and in vitro antibacterial evaluation of 2-hydroxy benzothiazole-based 1,3,4-oxadiazole derivatives
Source: Turk J Chem. 2020 Aug 18;44(4):1068–84. doi: 10.3906/kim-1912-55 (PMC7751938; doi:10.3906/kim-1912-55)
Supplement: Supplementary file 1 — Supplementary Materials [file turkjchem-44-1068-sup001.pdf]

<u>**SUPPLEMENTARY MATERIAL**</u>

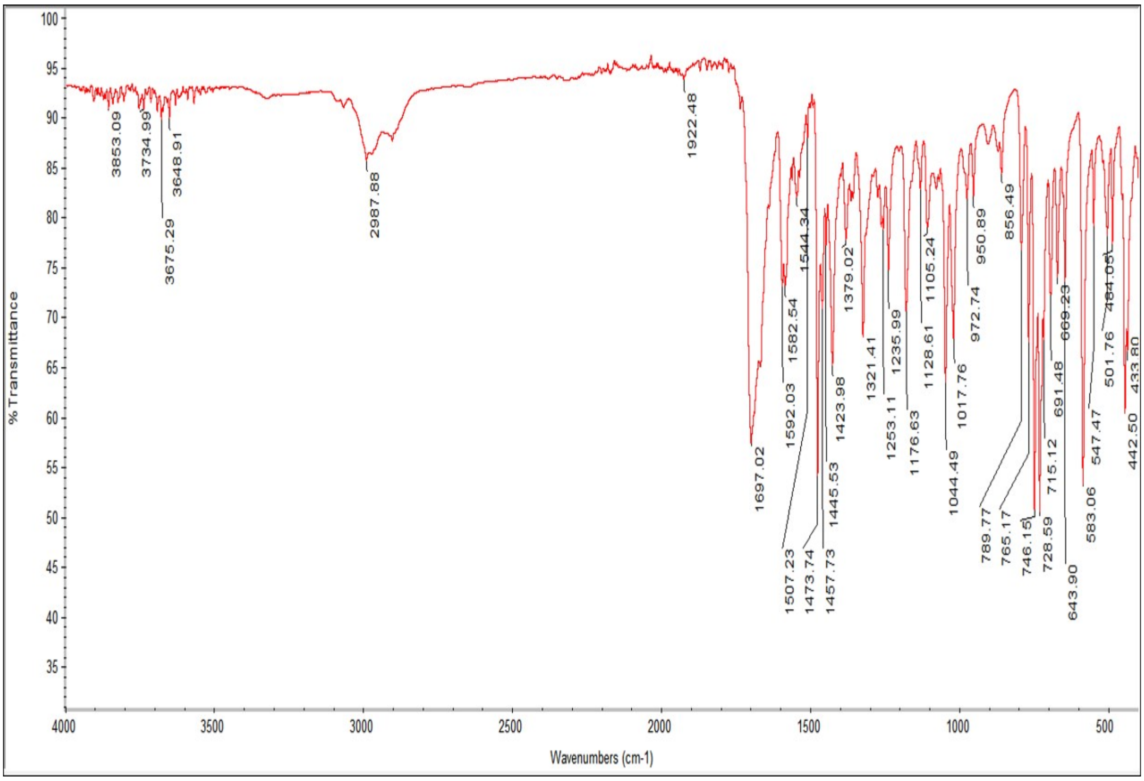

Figure S1. IR spectrum of compound **5**.

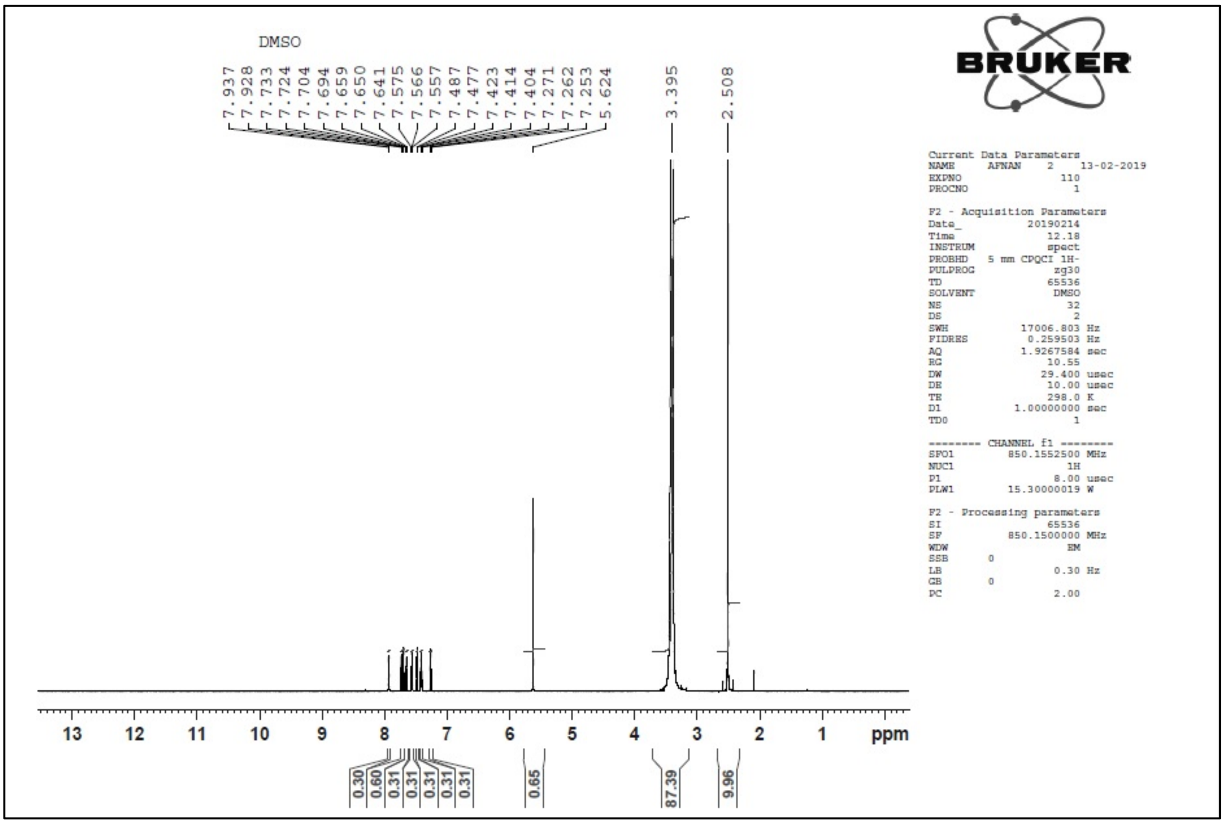

Figure S2. ^{1}H NMR spectrum of compound **5**.

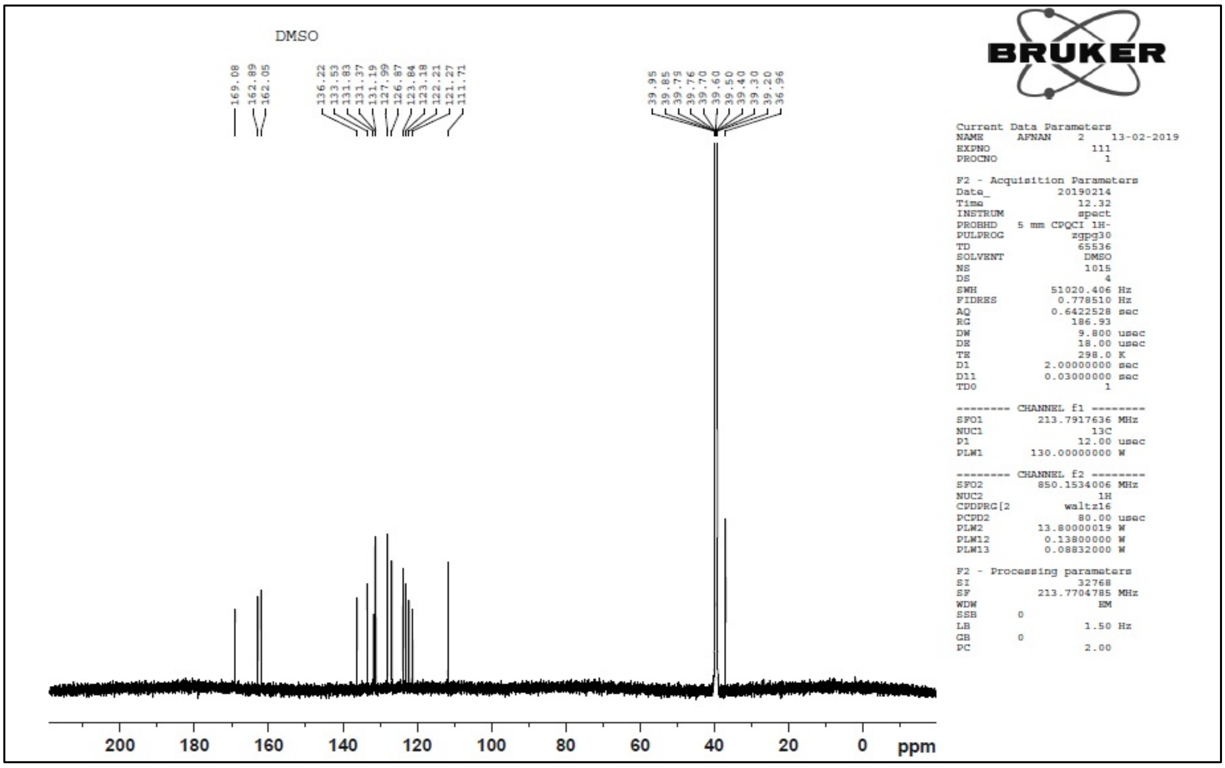

Figure S3. ^{13}C NMR spectrum of compound **5**.

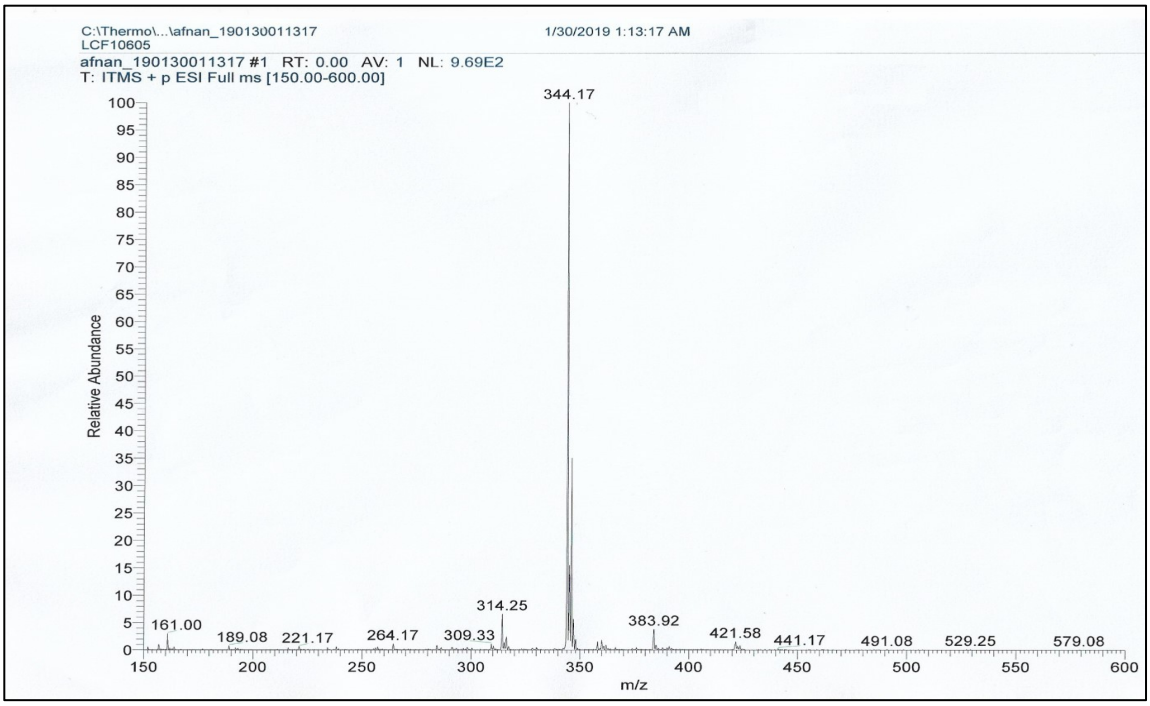

Figure S4. Mass spectrum of compound **5**.

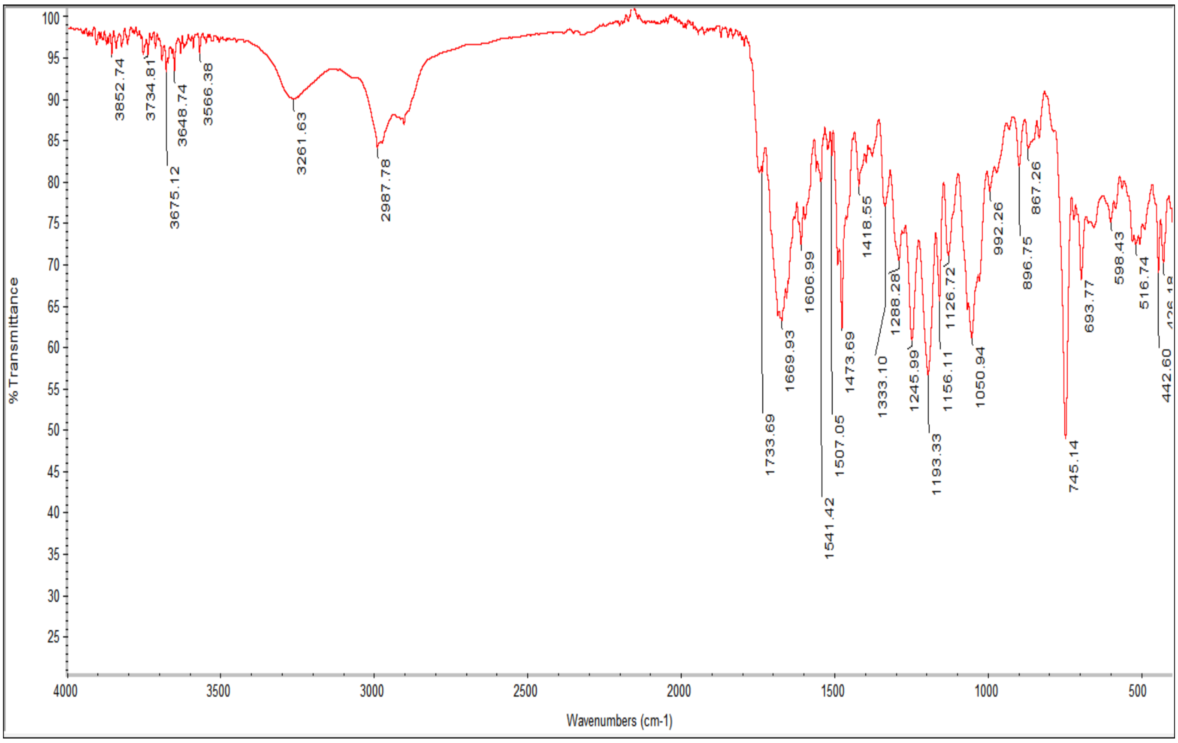

Figure S4. IR spectrum of compound **7**.

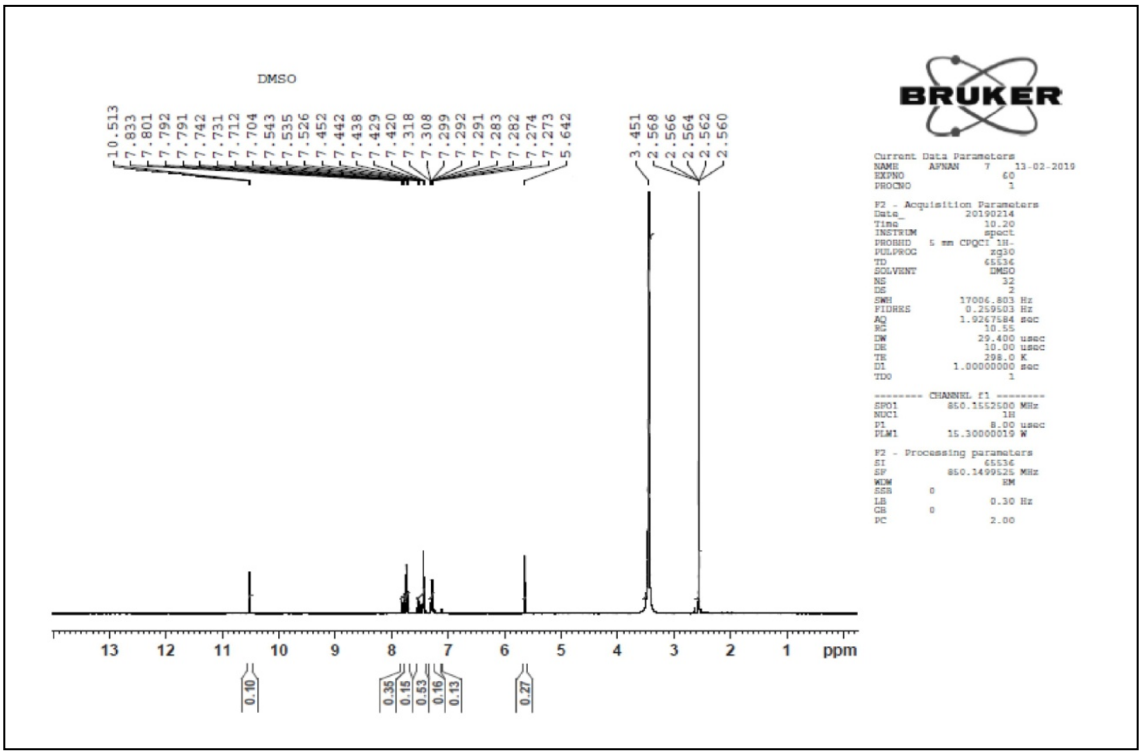

Figure S5. ^{1}H NMR spectrum of compound **7**.

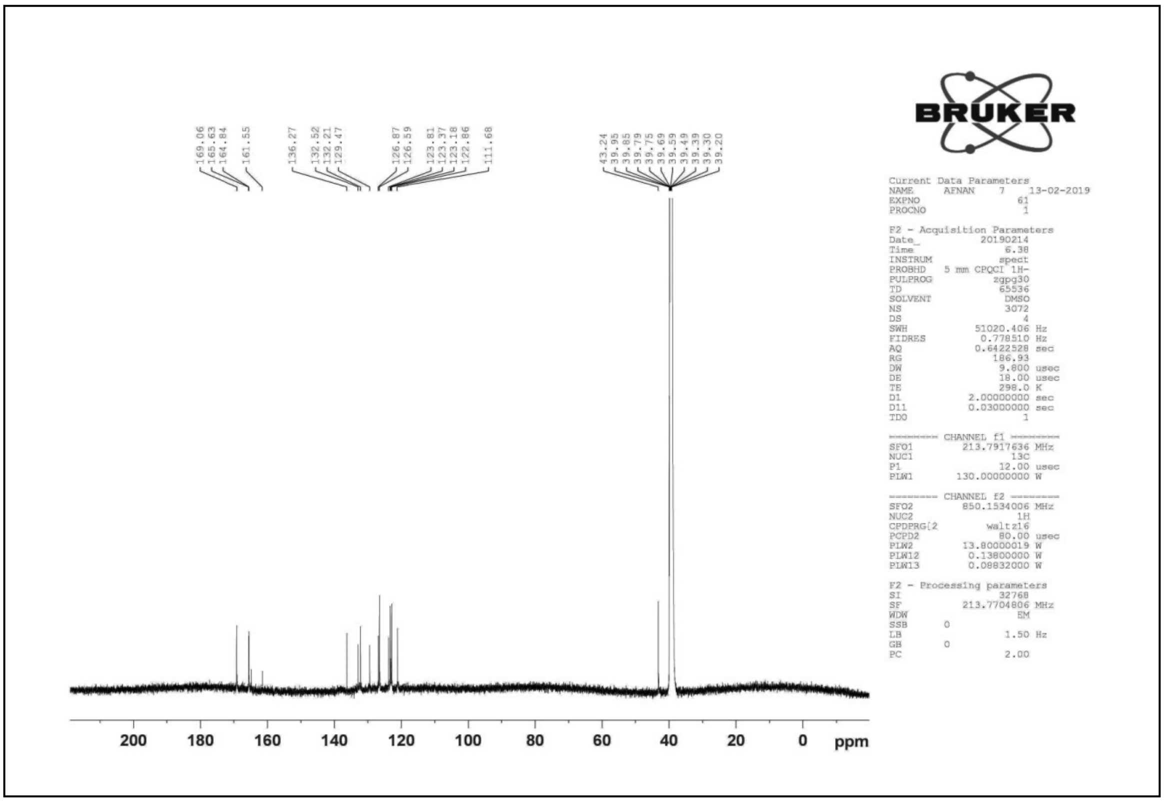

Figure S6. ^{13} C NMR spectrum of compound **7**.

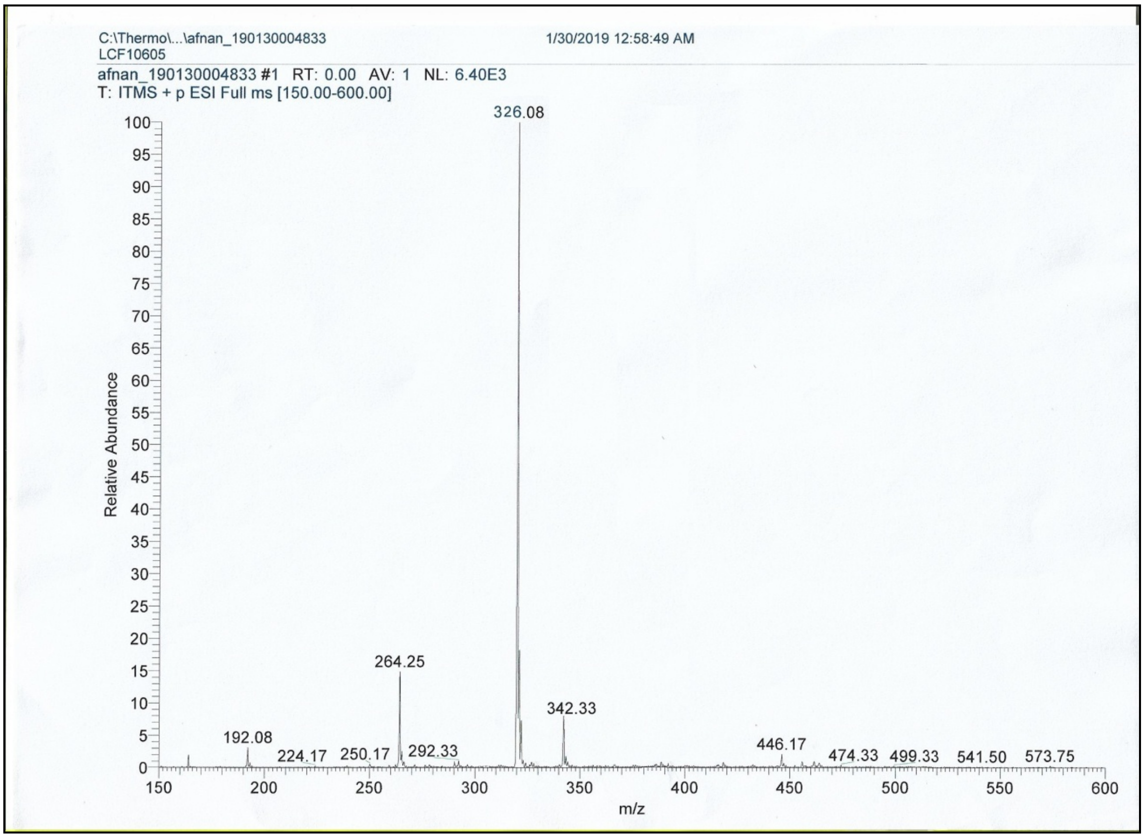

Figure S7. Mass spectrum of compound **7**.
